# Effectiveness of Lilly Connected Care Program (LCCP) App-Based Diabetes Education for Patients With Type 2 Diabetes Treated With Insulin: Retrospective Real-World Study

**DOI:** 10.2196/17455

**Published:** 2020-03-06

**Authors:** Yiyu Zhang, Chaoyuan Liu, Shuoming Luo, Jin Huang, Xia Li, Zhiguang Zhou

**Affiliations:** 1 Department of Metabolism and Endocrinology The Second Xiangya Hospital Central South University Changsha China; 2 Key Laboratory of Diabetes Immunology Ministry of Education Changsha China; 3 National Clinical Research Center for Metabolic Diseases Changsha China; 4 Department of Oncology The Second Xiangya Hospital Central South University Changsha China

**Keywords:** diabetes mellitus, mobile app, diabetes self-management education, glycemic control

## Abstract

**Background:**

Diabetes poses heavy economic and social burdens worldwide. Mobile apps show great potential for diabetes self-management education. However, there is limited evidence for the effectiveness of providing general diabetes education through mobile apps.

**Objective:**

The aim of this study was to clarify the effectiveness of Lilly Connected Care Program (LCCP) app-based diabetes education for glycemic control.

**Methods:**

This retrospective cohort study included patients with diabetes recruited to the LCCP platform from September 1, 2018, to May 31, 2019. Each patient was followed for 12 weeks. According to the number of diabetes education courses they had completed, the patients were divided into the following three groups: group A (0-4 courses), group B (5-29 courses), and group C (≥30 courses). The main outcomes were the change in blood glucose at the 12th week compared with baseline and the differences in blood glucose at the 12th week among the three groups. The associations of the number of diabetes education courses completed with the average blood glucose and frequency of self-monitoring of blood glucose (SMBG) at the 12th week were assessed by multivariate linear regression analyses controlling for other confounding covariates. Univariate and multivariate linear regression analyses were used to assess factors influencing patients’ engagement in the diabetes education courses.

**Results:**

A total of 5011 participants were enrolled. Their mean fasting blood glucose (FBG) and postprandial blood glucose (PBG) were significantly lower at the 12th week than at baseline (FBG, 7.46 [standard deviation (SD) 1.95] vs 7.79 [SD 2.18] mmol/L, *P*<.001; PBG, 8.94 [SD 2.74] vs 9.53 [SD 2.81] mmol/L, *P*<.001). The groups that completed more diabetes education courses had lower FBG (group B, β=−0.14, 95% CI −0.26 to −0.03; group C, β=−0.29, 95% CI −0.41 to −0.16; *P* for trend <.001) and PBG (group B, β=−0.29, 95% CI −0.46 to −0.11; group C, β=−0.47, 95% CI −0.66 to −0.28; *P* for trend <.001) and a higher frequency of SMBG at the 12th week (group B, β=1.17, 95% CI 0.81-1.53; group C, β=4.21, 95% CI 3.81-4.62; *P* for trend <.001) when compared with the findings in group A. Age and education were related to patients’ engagement in the diabetes education courses. Middle-aged patients (35-59 years old) and elderly patients (≥60 years old) completed more diabetes education courses (middle-aged group, β=2.22, *P*=.01; elderly group, β=2.42, *P*=.02) than young patients (18-34 years old).

**Conclusions:**

LCCP app-based diabetes education is effective for glycemic control and SMBG behavior improvement in patients with type 2 diabetes receiving insulin therapy. Young patients’ engagement in the education courses was relatively low. We need to conduct in-depth interviews with users to further improve the curriculum.

## Introduction

### Background

The prevalence of diabetes has been increasing worldwide [1,2]. It was estimated that there were 450 million adults with diabetes in 2017, and this figure is likely to increase to 690 million by 2045 [3]. According to a national survey in 2010, the prevalence of adult diabetes in China was 11.6%, representing more than 100 million adult patients with diabetes in China. Only 39.7% of those treated had ideal glycemic control [4]. Poor glycemic control can cause various complications, such as blindness, renal failure, and myocardial infarction [5]. In 2017, approximately 5 million adults died of diabetes [3]. In 2015, the global cost for diabetes was estimated to be approximately US $1.31 trillion [6].

Diabetes self-management and education are the cornerstones for diabetes management [7]. Studies have revealed that diabetes self-management education can help patients improve their glycemic control and self-management ability [8-10]. Diabetes guidelines also emphasize the importance of diabetes education [11,12]. In China, diabetes education traditionally takes the forms of group education classes in hospitals and individualized education in outpatient clinics. However, some patients do not have information on such classes, and it is inconvenient for them to take those classes because of distance and time constraints [13]. In addition, because medical resources in China are imbalanced, qualified diabetes educators are in short supply in rural areas. It may be difficult to provide high-quality diabetes education courses in primary hospitals [14]. Additionally, doctors from tertiary hospitals are overloaded with work and have limited time [15]. Outpatient consultations usually last only a few minutes. Patients receive little diabetes self-management knowledge in such a limited time [15]. Many patients with diabetes in China have not received any form of diabetes self-management education [16,17], and most Chinese patients with diabetes having poor glycemic control lack the ability to self-manage their diabetes [18].

Internet diabetes education is a potential way to overcome the barriers of distance, limited access, and short supply of qualified diabetes educators. Studies have also shown that internet-based diabetes education can improve glycemic control among patients with diabetes [19]. Mobile apps can receive and transmit information at any time and place. Compared with computers, mobile phones are easier to carry and easier to operate for the elderly [20]. With the popularity of smart phones, mobile apps have great potential for diabetes management. Many diabetes management apps provide general diabetes education for patients [21,22], and qualitative research has shown that for patients, gaining diabetes knowledge through mobile apps is more acceptable than receiving diabetes education classes in hospitals [23]. Studies have also suggested that diabetes management apps are beneficial for glycemic control and the self-management ability of patients with diabetes [24,25]. However, most studies have examined a mixture of remote monitoring, diabetes education, feedback from health care professionals, automatic feedback according to artificial intelligence, etc [21,26]. It is difficult to determine the functions of each feature [25,27,28]. Although feedback from health care professionals is important for the effectiveness of diabetes management apps [24,29,30], health care professionals are short on resources, and it is difficult to maintain their enthusiasm for using apps to manage patients, which are costly, without subsidies.

General diabetes education provided on mobile apps to patients with diabetes can make up for the shortage of diabetes educators to a certain degree. However, there is limited evidence that general diabetes education through mobile apps is effective for glycemic control among patients with diabetes. Studies have shown that the usage of diabetes apps varies across patients with different ages, education levels, and disease durations [31-33]. However, among patients who have already used apps to increase their diabetes knowledge, there is no relevant report on the difference in engagement across patients. Previous studies involving diabetes management apps were mostly randomized controlled trials with small samples. There is little real-world research on the effectiveness of diabetes management apps based on a large sample.

The Lilly Connected Care Program (LCCP) is a national diabetes care and support program that aims to improve diabetes management through internet technology and smart blood glucose monitoring devices with mobile communication for diabetes education and services in China. The LCCP is delivered via its official account on China’s largest social app WeChat. Patients can record their blood glucose levels, view their historical blood glucose records, and engage in diabetes education courses on the LCCP platform.

### Objectives

The aim of this study was to clarify the effectiveness of the LCCP app-based diabetes education program for glycemic control among patients with diabetes treated with insulin and to understand the factors associated with patients’ engagement in diabetes education on the LCCP platform.

## Methods

### Design and Sample

This was a retrospective cohort study that included patients with diabetes recruited to the LCCP platform from September 1, 2018, to May 31, 2019. Randomly selected outpatients with diabetes receiving insulin therapy (with or without oral hypoglycemic agents) from most major cities of 31 provinces in mainland China were encouraged by their physicians to register on the LCCP platform without any financial incentives. After informed consent was obtained, patient demographic information, such as gender, age, education, type of diabetes, insulin regimen, and duration of diabetes, was collected. Each patient was followed up for 12 weeks. Eligible participants were patients with fasting blood glucose (FBG) and postprandial blood glucose (PBG) records on the platform at least once a week at week 1 and week 12. Patients with type 1 diabetes, patients aged <18 years, and patients with missing data on gender, age, education, and duration of diabetes were excluded from the study.

### Intervention

There are 60 diabetes education courses on the LCCP platform. Patients can choose the education courses of interest on the LCCP platform to learn. Each course includes 1-5 sections, and each section takes 5-10 minutes to complete. The courses are presented in the form of audio and text. There is a small quiz after the completion of each section to test and consolidate patients’ diabetes knowledge. Only when all the questions are answered correctly can the patient view the next section. Completing all 60 courses requires about 13 hours in total. The courses on the LCCP platform were created by experts in accordance with the standards of medical care for type 2 diabetes in China [12]. The courses cover patients’ self-care behaviors according to the American Association of Diabetes Educators 7 Standard of Care [34], including eating healthy, being active, monitoring glucose, taking medications, solving problems, coping in healthy ways, and reducing risks. These courses enable patients to fully understand the necessity of glycemic control and the harm of diabetic complications. In addition to providing knowledge, self-management behavior change strategies are included (see Multimedia Appendix 1 for more details).

### Outcome Measurements

Recruited patients were provided with a blood glucose monitoring kit that included a free intelligent glucometer and test strips. Self-monitoring of finger-prick capillary blood glucose was tested according to the glucose dehydrogenase method using an intelligent glucometer (Bionime Biotechnology [Ping Tan] Co, Ltd, Fuzhou City, China). The patients were taught to measure their FBG and PPG correctly to reduce subject bias. Data regarding patients’ self-monitoring of blood glucose (SMBG) was automatically uploaded to the app platform through mobile 3G signals. The coefficient of variation of the measurement was below 5%, and the accuracy was in accordance with ISO 15197:2013 [35]. The baseline FBG and PBG were defined as the mean FBG and the mean PBG at the first week after recruitment. According to the number of diabetes education courses completed, the patients were divided into the following three groups: group A (0-4 courses), group B (5-29 courses), and group C (≥30 courses). Because the number of patients who did not complete any education courses was very small, we grouped the patients who completed 0 to 4 courses into one group and considered these patients as having the least engagement in the diabetes education courses. The remaining patients were divided into two groups according to whether they completed more than half of the total courses. The main outcomes were the change in the mean FBG and mean PBG at the 12th week compared with baseline and the difference in the mean FBG and mean PBG at the 12th week among the three groups. The secondary outcomes were the relationships between the number of diabetes education courses completed and the frequency of SMBG, as well as the factors associated with patients’ engagement in the diabetes education courses. We defined patients’ engagement in the diabetes education courses as the number of diabetes education courses that the patients completed.

### Ethics

All patients provided written informed consent when recruited to the LCCP platform. This study was approved by the ethics committee of the Second Xiangya Hospital, Central South University.

### Statistical Analysis

All continuous variables with a normal distribution are presented as means (standard deviations [SDs]). Variables with a nonnormal distribution are presented as medians (IQRs). Categorical variables are presented as the frequency (number of cases [n]) and percentage (%) of total study patients. A paired *t*-test was used to assess the change in blood glucose from baseline to week 12. For intragroup comparisons, an analysis of variance (ANOVA) test was used. The associations of the number of diabetes education courses completed with the average blood glucose and SMBG at the 12th week were assessed using multivariate linear regression analysis while controlling for other confounding covariates. To evaluate linear trends, we entered the median level of the diabetes education courses completed by category into the model as a continuous variable [36-38]. Univariate and multivariate linear regression analyses were used to assess the factors influencing patients’ engagement in the diabetes education courses. Statistical analyses were performed using SAS 9.4 software (SAS Institute, Cary, North Carolina) via SAS Enterprise Guide version 7.1. A *P* value and *P* for trend ≤.05 were considered statistically significant.

## Results

### Patient Characteristics

**Figure 1:**
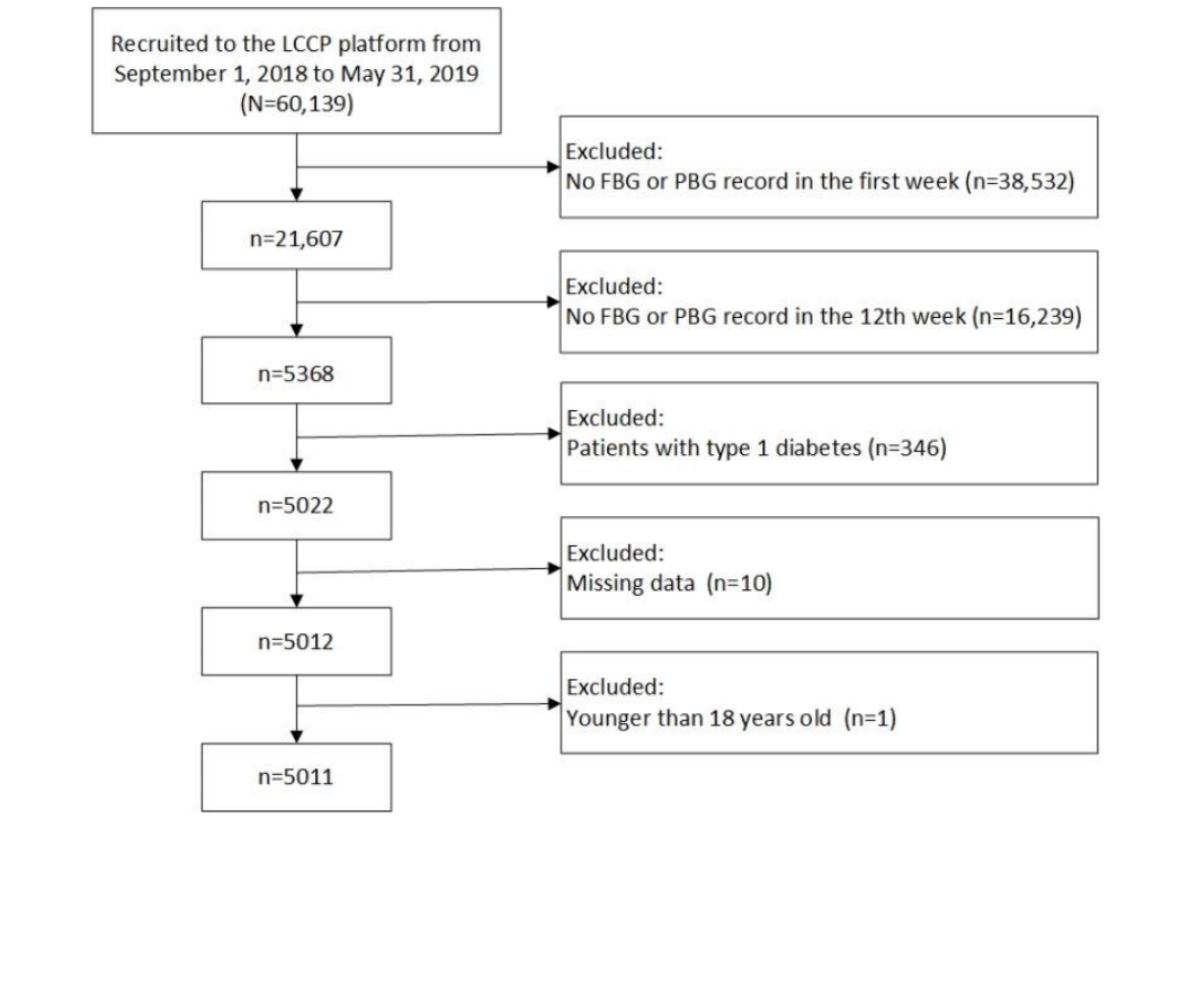
The patient inclusion flow chart. FBG: fasting blood glucose; LCCP: Lilly Connected Care Program; PBG: postprandial blood glucose.

From September 1, 2018, to May 31, 2019, a total of 5011 patients with type 2 diabetes who were older than 18 years and were receiving insulin therapy were enrolled in the study. The patient inclusion flow chart is shown in Figure 1. The sample was recruited from 31 provinces across China. Among the 5011 patients, 56.02% (2807/5011) were male, and the median age was 52.0 years (IQR 43.0-60.0 years). The median duration of diabetes was 2.25 years (IQR 0.08-9.50 years). Patient characteristics at baseline are shown in Table 1.

### Effect on Glycemic Control

The mean FBG at baseline was 7.79 (SD 2.18) mmol/L, and the mean PBG was 9.53 (SD 2.81) mmol/L. The mean FBG and PBG of the patients were significantly lower at the 12th week than at baseline (FBG, 7.46 [SD 1.95] vs 7.79 [SD 2.18] mmol/L, *P*<.001; PBG, 8.94 [SD 2.74] vs 9.53 [SD 2.81] mmol/L, *P*<.001). Among the patients, 63.10% (3162/5011) had poor baseline glycemic control (FBG ≥7 mmol/L or PBG ≥11 mmol/L). Among the patients with poor baseline blood glucose values, both FBG and PBG at the 12th week were significantly decreased from baseline (FBG, 8.02 [SD 2.04] vs 8.83 [SD 2.07] mmol/L, *P*<.001; PBG, 9.48 [SD 2.93] vs 10.52 [SD 2.93] mmol/L, *P*<.001).

**Table 1:** Patient characteristics at baseline (N=5011).

Variable		Value^a^
Duration of diabetes (years)		2.25 (0.08-9.50)
Age (years)		52.0 (43.0-60.0)
**Age group (years)**		
	18-34	480 (9.58)
	35-59	3157 (63.00)
	≥60	1374 (27.42)
**Gender**		
	Male	2807 (56.02)
	Female	2204 (43.98)
**Education**		
	Junior middle school or below	1372 (27.38)
	High school	1644 (32.81)
	College or above	1995 (39.81)
**Insulin regimen**		
	Premixed insulin	3909 (78.0)
	Fast-acting insulin (with/without long-acting insulin)	1102 (21.99)
**Region**		
	Northeast China	963 (19.22)
	North China	972 (19.40)
	East China	1395 (27.84)
	South China	400 (7.98)
	Central China	733 (14.63)
	Northwest China	248 (4.95)
	Southwest China	300 (5.99)

^a^Continuous data are presented as medians (interquartile ranges), and categorical data are presented as n (%).

According to the number of diabetes education courses completed, the patients were divided into the following three groups: group A (0-4 courses), group B (5-29 courses), and group C (≥30 courses). The mean FBG and PBG of each group decreased compared with baseline (all *P*<.001; Table 2).

Among the three groups, the mean FBG and PBG were lower in group C than in group B and group A at the 12th week (FBG, 7.28 [SD 1.87] vs 7.44 [SD 1.91] vs 7.67 [SD 2.08] mmol/L, *P*<.001; PBG, 8.70 [SD 2.54] vs 8.91 [SD 2.65] vs 9.26 [SD 3.06] mmol/L, *P*<.001). After adjusting for multiple confounding factors, including gender, age, education, duration of diabetes, baseline insulin regimen, and baseline FBG and PBG, multivariate linear regression analysis showed that the number of diabetes education courses completed was still related to both FBG and PBG (Table 3). Patients in groups B and C, who completed more diabetes education courses, had lower mean FBG (group B, β=−0.14, 95% CI −0.26 to −0.03; group C, β=−0.29, 95% CI −0.41 to −0.16; *P* for trend <.001) and PBG at the 12th week (group B, β=−0.29, 95% CI −0.46 to −0.11; group C, β=−0.47, 95% CI −0.66 to −0.28; *P* for trend <.001) compared with the findings for patients in group A (the lowest number of diabetes education courses completed).

**Table 2:** Comparison of mean blood glucose at week 12 and baseline.

Group	Participants, n (%)	Baseline FBG^a^ (mmol/L), mean (SD)	FBG (mmol/L), mean (SD)	*P* value	Baseline PBG^b^ (mmol/L), mean (SD)	PBG (mmol/L), mean (SD)	*P* value
Total	5011 (100)	7.79 (2.18)	7.46 (1.95)	<.001	9.53 (2.81)	8.94 (2.74)	<.001
Group A	1328 (26.5)	7.93 (2.26)	7.67 (2.08)	<.001	9.64 (3.08)	9.26 (3.06)	<.001
Group B	2258 (45.1)	7.76 (2.15)	7.44 (1.91)	<.001	9.56 (2.75)	8.91 (2.65)	<.001
Group C	1425 (28.4)	7.69 (2.15)	7.28 (1.87)	<.001	9.37 (2.64)	8.70 (2.54)	<.001

^a^FBG: fasting blood glucose.

^b^PBG: postprandial blood glucose.

**Table 3:** The relationship between mean blood glucose at the 12th week and the number of diabetes education courses completed according to linear regression analysis.

Outcome and group		Crude model						Adjusted model^a^					
			β (95% CI)		*P* value		*P* value for trend			β (95% CI)	*P* value		*P* value for trend
**FBG^b^** (12th week)****													
	Group A^c^		—^d^		—		<.001			—	—		<.001
	Group B		−0.23 (−0.36 to −0.10)		<.001					−0.14 (−0.26 to −0.03)	.01		
	Group C		−0.39 (−0.54 to −0.25)		<.001					−0.29 (−0.41 to −0.16)	<.001		
**PBG^e^ (12th week)**													
	Group A^c^		—		—		<.001			—	—		<.001
	Group B		−0.34 (−0.53 to −0.16)		<.001					−0.29 (−0.46 to −0.11)	.001		
	Group C		−0.56 (−0.76 to −0.35)		<.001					−0.47 (−0.66 to −0.28)	<.001		

^a^Adjusted for gender, age, education, duration of diabetes, baseline insulin regimen, and baseline FBG and PBG.

^b^FBG: fasting blood glucose.

^c^Reference group.

^d^Not applicable.

^e^PBG: postprandial blood glucose.

### Effect on Self-Monitoring of Blood Glucose Behavior

The mean frequency of SMBG at the 12th week was 7.95 (SD 5.79) times per week. Among the three groups, the frequency of SMBG was higher in group C than in group B and group A at the 12th week (10.97 [SD 7.06] vs 7.39 [SD 4.92] vs 5.67 [SD 4.00] times per week, *P*<.001). After adjusting for multiple confounding factors, including gender, age, education, duration of diabetes, baseline FBG and PBG, and baseline SMBG frequency, multivariate linear regression analysis showed that the number of diabetes education courses that patients completed was still related to the frequency of SMBG at the 12th week (Table 4). Patients in groups B and C, who completed more diabetes education courses, had higher frequencies of SMBG at the 12th week (group B, β=1.17, 95% CI 0.81-1.53; group C, β=4.21, 95% CI 3.81-4.62; *P* for trend <.001) compared with the findings for patients in group A (the lowest number of diabetes education courses completed).

**Table 4:** The relationship between self-monitoring of blood glucose frequency at the 12th week and the number of diabetes education courses completed according to linear regression analysis.

Group	SMBG^a^ frequency (times per week), mean (SD)	Crude model			Adjusted model^b^		
		β (95% CI)	*P* value	*P* value for trend	β (95% CI)	*P* value	*P* value for trend
Group A^c^	5.67 (4.01)	—^d^	—	<.001	—	—	<.001
Group B	7.39 (4.92)	1.72 (1.35-2.09)	<.001		1.17 (0.81-1.53)	<.001	
Group C	10.97 (7.06)	5.30 (4.9-5.71)	<.001		4.21 (3.81-4.62)	<.001	

^a^SMBG: self-monitoring of blood glucose.

^b^Adjusted for gender, age, education level, duration of diabetes, baseline SMBG frequency, fasting blood glucose, and postprandial blood glucose.

^c^Reference group.

^d^Not applicable.

### Factors Influencing Engagement

The median number of diabetes education courses completed in 12 weeks was 14 (IQR 4-33). Univariate linear regression analysis showed that age (middle-aged patients, β=2.29, *P*=.01; elderly patients, β=2.72, *P*=.005), education (high school, β=1.40, *P*=.04), and baseline FBG (β=−0.27, *P*=.02) and PBG (β=−0.21, *P*=.02) were related to the number of diabetes education courses completed. Gender (*P*=.24) and duration of diabetes (*P*=.10) did not show such an association. After mutual adjustment by multivariate linear regression, the age and education level of patients were still related to the number of diabetes education courses completed (Table 5). Compared with young patients (18-34 years old), middle-aged patients (35-59 years old) and elderly patients (≥60 years old) completed more diabetes education courses (middle-aged group, β=2.22, *P*=.01; elderly group, β=2.42, *P*=.02). Compared with patients having a junior middle school education or below, the number of diabetes education courses completed was higher among patients having a high school education (β=1.46, *P*=.03) but was not significantly different among patients having a college education or above (*P*=.98).

**Table 5:** Factors associated with the number of diabetes education courses completed according to linear regression analysis.

Variables		Univariate model	*P* value	Multivariate model	*P* value
		β (95% CI)		β (95% CI)	
**Gender**					
	Male^a^	—^b^	—	—	—
	Female	0.60 (−0.40 to 1.61)	.24	0.61 (−0.41 to 1.62)	.24
**Age (years)**					
	18-34^a^	—	—	—	—
	35-59	2.29 (0.55 to 4.03)	.01	2.22 (0.46 to 3.98)	.01
	≥60	2.72 (0.84 to 4.60)	.005	2.42 (0.43 to 4.41)	.02
**Education**					
	Junior middle school or below^a^	—	—	—	—
	High school	1.40 (0.10 to 2.70)	.04	1.46 (0.16 to 2.75)	.03
	College or above	−0.26 (−1.51 to 0.98)	.68	−0.02 (−1.28 to 1.24)	.98
Duration of diabetes		0.06 (−0.01 to 0.12)	.10	0.05 (−0.02 to 0.12)	.15
Baseline FBG^c^		−0.27 (−0.50 to −0.04)	.02	−0.22 (−0.49 to 0.05)	.11
Baseline PBG^d^		−0.21 (−0.39 to −0.03)	.02	−0.18 (−0.38 to 0.03)	.10

^a^Reference group.

^b^Not applicable.

^c^FBG: fasting blood glucose.

^d^PBG: postprandial blood glucose.

## Discussion

### Principal Findings

Diabetes management apps are beneficial for glycemic control in patients with diabetes. However, the results of different studies showed great heterogeneity, which might be related to the different features of apps. Most studies examined a mixture of telemonitoring, education, feedback, and other functions. It was not clear which function played a role [27]. A study by Dong et al found that health education via WeChat in conjunction with conventional diabetes treatment could improve glycemic control in patients with type 2 diabetes. Because the intervention was not blinded, a certain degree of collaborative intervention might exist [39]. Our study showed that diabetes education courses on the mobile app-based LCCP platform were helpful for improving glycemic control in patients with diabetes treated with insulin. After 3 months of obtaining diabetes self-management knowledge on the LCCP platform, both the FBG and PBG of patients decreased.

The use frequency of diabetes management apps varies across different patients. Most studies did not explore the effect of app use frequency on glycemic control. A study by Agarwal et al found that a diabetes management app did not affect patients’ diabetes management. A possible reason was the low use of the app among participants [40]. Vehi et al found a relevant effect of a diabetes management app on blood glucose, independent of the use frequency [41]. By recording the number of diabetes education courses that the patients completed, our study found that completion of more diabetes education courses by patients was associated with more improvement in their glycemic control. Previous studies showed that the effect of diabetes education was related to age, duration of diabetes, and education level [7,42]. After adjusting for multiple confounding factors, including gender, age, education, duration of diabetes, baseline insulin regimen, and baseline FBG and PBG, the number of diabetes education courses that the patients completed was still related to their glycemic control. A systematic review showed that diabetes self-management education can improve the glycemic control of patients, and the effect was higher for interventions that offered more than 10 contact hours [43]. This finding is consistent with our finding. A qualitative study found that existing diabetes education programs may not adequately meet all the needs of patients with type 2 diabetes [44]. Offline diabetes education classes need diabetes educator resources, and there are many barriers preventing patients from taking education classes in hospitals regularly [13,45]. No in-person diabetes education was provided in our study. Although individualized diabetes education is emphasized [46], app-based general diabetes education can save resources, is convenient for patients, and is inexpensive. It can be used as a supplement to traditional diabetes education and can mitigate the deficiencies of traditional diabetes education to some extent.

By comparing the frequency of SMBG in patients with different numbers of diabetes education courses completed, we found that the frequency of SMBG was related to the completion of diabetes education courses. After adjusting for multiple confounding factors, we found that completion of more diabetes education courses by patients was associated with a higher frequency of SMBG. Although the role of SMBG is controversial in patients with type 2 diabetes treated with oral agents, SMBG in patients with type 2 diabetes treated with insulin is very important; it is useful for adjusting insulin dosage, guiding nutrition therapy and physical activity, and preventing hypoglycemia [11,47]. The difference in the glycemic control improvement of patients with different numbers of diabetes education courses completed may partly be related to differences in their SMBG behavior. However, to be useful, SMBG information needs to be integrated with patients’ clinical and self-management plans [47]. Therefore, if blood glucose monitoring data on the app platform are supplemented with feedback from health care professionals, the effect of the app might be stronger.

Previous studies showed that diabetes education can improve patients’ glycemic control and self-management behaviors [7,10,48]. According to the health belief model and extended unified theory of acceptance and use of technology, patients’ belief in health risk and perceived usefulness can predict the likelihood of their engagement in health behaviors [49]. The diabetes education courses on the LCCP platform emphasize the necessity of glycemic control and the harm of diabetes complications. After completing the courses, patients might realize the harm of diabetes and the usefulness of glycemic control and improve their self-care behaviors. Strategies for improving self-management behaviors are also provided in the LCCP education courses. These might be the possible mechanisms by which the educational content on the LCCP platform led to behavior changes and glycemic control improvement.

Our study not only identified the effect of diabetes education courses but also analyzed the factors influencing patients’ engagement in these courses. We found that patients’ age and education were related to their engagement in diabetes education courses. A previous study found that women and patients with a higher education status were more willing to participate in offline diabetes education than men and patients with a lower education status [50]. However, our study did not find gender differences. Previous studies found that health app usage was higher in younger patients than in older patients [51,52]. However, our study found that among patients who had already used the app, middle-aged and elderly patients were more involved than younger patients. The reasons for refusing to participate in diabetes education include good overall wellbeing and individuals’ sense that they can manage their disease and that they have sufficient knowledge [7]. Our previous study found that effort expectancy had only a slight effect on patients’ willingness to use diabetes apps [53]. With the widespread use of smart phones among the elderly, they may not experience difficulty operating such apps. Additionally, young patients may have less time to learn because they may have to work, may have other channels through which to gain diabetes knowledge, or may have a higher self-efficacy in diabetes management, which may explain why they engaged less in diabetes education courses on the app. Perceived usefulness is the most important determinant of patients’ intention to use diabetes apps [54]. We should conduct in-depth interviews with users to further identify the factors that affect patients’ perceived usefulness of the education courses to further improve the curriculum.

### Strengths and Limitations

Most studies on the effectiveness of diabetes apps and diabetes education programs were not blinded randomized controlled trials and had small samples. A certain degree of performance bias might be unavoidable. Our study was based on real-world data with a large sample. By comparing the differences in glycemic control between patients with different engagement levels in diabetes education courses, we found that diabetes education on the app platform was effective in diabetes management among patients with type 2 diabetes treated with insulin.

However, our study had several limitations. First, our observation period was short. The long-term effect of diabetes education provided through the app needs to be further investigated, and future studies could adopt glycosylated hemoglobin as an indicator of blood glucose levels. In addition, we did not collect other information, such as rural or urban residence, social-economic status, and chronic complications. These factors may have some impact on glycemic control and patients’ participation in diabetes education courses. Finally, we found that the LCCP app-based diabetes education was associated with improvement in glycemic control, but it was unclear whether the improvement in glycemic control was due to improvements in patients’ self-management behaviors. Self-management behaviors include a healthy diet, regular physical activity, SMBG, and medication adherence [34]. Our study observed improvements only in SMBG behavior; improvements in diet and exercise behaviors and drug compliance need to be further investigated.

### Conclusions

LCCP app-based diabetes education is effective for glycemic control and improvement in SMBG behavior among patients with type 2 diabetes receiving insulin therapy. Young patients’ engagement in these education courses is relatively low. We need to conduct in-depth interviews with users to identify the factors that affect patients’ perceived usefulness of the education courses to further improve the curriculum.

## Appendix

Multimedia Appendix 1 Main contents of the Lilly Connected Care Program diabetes education courses.

## Abbreviations

FBG: fasting blood glucose
LCCP: Lilly Connected Care Program
PBG: postprandial blood glucose
SMBG: self-monitoring of blood glucose
