# Supplementary material for: Effectiveness of Lilly Connected Care Program (LCCP) App-Based Diabetes Education for Patients With Type 2 Diabetes Treated With Insulin: Retrospective Real-World Study
Source: JMIR Mhealth Uhealth. 2020 Mar 6;8(3):e17455. doi: 10.2196/17455 (PMC7084288; doi:10.2196/17455)
Supplement: Multimedia Appendix 1 [file mhealth_v8i3e17455_app1.docx]

**Appendix 1:** Main contents of the LCCP diabetes education courses

| Catalogues | Main contents |
| --- | --- |
| Insulin injection | Includes 7 courses covering introduction of insulin, insulin injection method, insulin dose adjustment, prevention of hypoglycemia, coping strategies for insulin injection associated complications, etc. |
| Healthy diet | Includes 13 courses covering the association of a healthy diet and glycemic control, strategies for controlling postprandial blood glucose, medical nutrition therapy, etc. |
| Exercise therapy | Includes 6 courses covering the association of exercise therapy and glycemic control, choice of exercise types, strategies for prevention of hypoglycemia and other risks during exercise, etc. |
| Lifestyle change | Includes 6 courses covering alcohol intake, blood glucose control during traveling，smoking cessation, the importance of lifestyle change in diabetes patients, etc. |
| Monitoring | Includes 11 courses covering the correct use of a blood glucose meter, monitoring and prevention of hypoglycemia, complication monitoring, individualized self-monitoring of blood glucose plan, hemoglobin A1c, recognition of the importance of blood glucose self-monitoring, strategies for adjusting blood glucose according to the monitoring of blood glucose, etc. |
| Medication | Includes 6 courses covering the importance of medication adherence, remedies when forgetting to take medicine, introduction of hypoglycemic drugs, etc. |
| Complication prevention | Includes 9 courses covering an introduction of acute and chronic diabetes complications, screening of diabetes complications, prevention and control of diabetic complications, etc. |
| Etiology of diabetes | Includes 2 courses introducing the introduction of diabetes, and associations of diabetes and heredity |
